# Blood amyloid and tau biomarkers as predictors of cerebrospinal fluid profiles

**DOI:** 10.1007/s00702-022-02474-9

**Published:** 2022-02-15

**Authors:** Constance Delaby, Daniel Alcolea, Christophe Hirtz, Jérôme Vialaret, Jana Kindermans, Lisa Morichon, Juan Fortea, Olivia Belbin, Audrey Gabelle, Kaj Blennow, Henrik Zetterberg, Alberto Lleó, Sylvain Lehmann

**Affiliations:** 1 Laboratoire de Biochimie Protéomique Clinique (LBPC-PPC), Univ Montpellier, CHU Montpellier, INM INSERM, Hôpital St Eloi, IRMB 80 av A Fliche, 34295 Montpellier, France; 2 Hospital de la Santa Creu i Sant Pau, Biomedical Research Institute Sant Pau, Universitat Autònoma de Barcelona, Barcelona, Spain; 3 CMRR, Univ Montpellier, CHU Montpellier, INM INSERM, Montpellier, France; 4 Department of Psychiatry and Neurochemistry, Institute of Neuroscience and Physiology, The Sahlgrenska Academy at the University of Gothenburg, Gothenburg, Sweden; 5 Clinical Neurochemistry Laboratory, Sahlgrenska University Hospital, Mölndal, Sweden; 6 Department of Neurodegenerative Disease, UCL Institute of Neurology, Queen Square, London, UK; 7 UK Dementia Research Institute at UCL, London, UK; 8 Hong Kong Center for Neurodegenerative Diseases, Hong Kong, China

**Keywords:** Blood, Biomarkers, Clinical management, Lumbar puncture, CSF

## Abstract

**Introduction:**

Blood biomarkers represent a major advance for improving the management, diagnosis, and monitoring of Alzheimer's disease (AD). However, their context of use in relation to routine cerebrospinal fluid (CSF) analysis for the quantification of amyloid peptides and tau proteins remains to be determined.

**Methods:**

We studied in two independent cohorts, the performance of blood biomarkers in detecting “nonpathological” (A−/T−/N−), amyloid (A+) or neurodegenerative (T+ /N+) CSF profiles.

**Results:**

Plasma Aβ_1–42_/Aβ_1–40_ ratio and phosphorylated tau (p-tau(181)) were independent and complementary predictors of the different CSF profile and in particular of the nonpathological (A−/T−/N−) profile with a sensitivity and specificity close to 85%. These performances and the corresponding biomarker thresholds were significantly different from those related to AD detection.

**Conclusion:**

The use of blood biomarkers to identify patients who may benefit from secondary CSF testing represents an attractive stratification strategy in the clinical management of patients visiting memory clinics. This could reduce the need for lumbar puncture and foreshadow the use of blood testing on larger populations.

**Supplementary Information:**

The online version contains supplementary material available at 10.1007/s00702-022-02474-9.

## Introduction

Detection of Alzheimer's disease (AD) with high sensitivity and specificity is key for the management of patients. Diagnosis can include detection of amyloid and tau biomarkers in the cerebrospinal fluid (CSF), which is one of international guidelines’ criteria (Dubois et al. 2014; McKhann et al. 2011). Thus, the identification of AD processes years before the onset of symptoms recently triggered a paradigm shift in which AD could be viewed as a biological rather than clinical entity (Jack et al. 2018). The importance of biomarkers was also emphasized when defining the unbiased "A/T/N" classification system (Jack et al. 2016). These evolutions are coupled with the prospect of introducing treatments that would modify the trajectory of the disease by delaying its clinical expression. However, the use of CSF to detect AD at an early stage in a large population remains difficult because of the invasive nature of lumbar puncture (LP). Blood biomarkers are in this context, of particular interest. The possibility of detecting amyloid peptides and tau proteins in plasma has recently shaken the field of neurodegenerative diseases detection. Many research groups, including ours, are evaluating the diagnostic value of these biomarkers for the accurate detection of AD, through cross sectional or longitudinal studies using retrospective samples (Alcolea et al. 2021; Lewczuk et al. 2018; Brickman et al. 2021).

However, one context of use (COU) that has not yet been directly addressed is in relation to CSF testing performed in a routine clinical setting. The question is whether blood biomarkers can be used to decide the need for further CSF analysis. Thus, the objective here is to evaluate the performance of blood biomarkers in detecting a “nonpathological” (A−/T−/N−) CSF profile, rather than a specific pathological profile as seen in AD or brain injury. Note that the notion of “nonpathological” does not refer here to the globality of the CSF analysis which includes many other biochemical, immunological or microbiological analyses, but is restricted to the results of the amyloid and tau biomarkers. The results of this study suggest that blood markers can predict the presence of nonpathological CSF profile and could thus be decisive in whether or not to perform a LP.

## Methods

### Participants

The Barcelona cohort included 150 participants from the Sant Pau Initiative on Neurodegeneration (SPIN cohort) (Alcolea et al. 2019) evaluated at the Sant Pau Memory Unit (Barcelona, Spain) between November 2013 and October 2019. Participants in this cohort mostly were patients with a diagnosis of AD, Dementia with Lewy bodies (DLB), frontotemporal lobar degeneration-related syndromes (FTLD), and mild cognitive impairment (MCI), or cognitively normal controls. The two cohorts differ in the distribution of A/T/N profiles based on CSF biomarkers, with the Barcelona cohort having a much higher percentage of isolated amyloid-positive patients (A+/N−/T−) than the Montpellier cohort (Table 1). All participants had received neurological and neuropsychological evaluation and provided CSF and plasma samples. The Montpellier cohort included 161 patients recruited from September 2009 to June 2017 (Lehmann et al. 2020). All patients underwent a thorough clinical examination including biological laboratory tests, neuropsychological assessments, and brain imaging. In addition to AD, DLB, FTLD and MCI, patient from this cohort had also mixed dementia, normal pressure hydrocephalus and Parkinson disease with cognitive signs or subjective cognitive impairment.

**Table 1:** Demography, CSF biomarker values and AT(N) classification of the cohort of Montpellier and Barcelona

Variable	The Montpellier cohort		The Barcelona cohort	
	Mean	SD	Mean	SD
Age (years)	68.3	10.5	67.6	12.3
Sex (M%)*	60.3	–	39.4	–
CSF biomarkers				
Aβ_1–40_ (pg/mL)	15,940	6875	12,078	3838
Aβ_1–42_ (pg/mL)	826	377	906	446
Tau (pg/mL)	461	320	467	326
p-tau(181) (pg/mL)	66	43	73	63
ATN*				
A−/T−/N−	38.1%	–	31.8%	–
A−/T−/N+	4.8%	–	3.9%	–
A−/T+/N−	1.6%	–	0.6%	–
A−/T+/N+	4.8%	–	2.6%	–
A+/T−/N−	4.8%	–	25.8%	–
A+/T−/N+	3.2%	–	0,6%	–
A+/T+/N−	4.8%	–	3.9%	–
A+/T+/N+	38.1%	–	31.1%	–

*SD* standard deviation

^*^Significant difference

All participants gave their written informed consent to participating in clinical research on CSF and plasma biomarkers, and protocols at both centers were approved by the respective Ethics Committees.

### Primary outcomes

CSF Aβ_1–42_, Aβ_1–40_, total tau and phosphorylated tau 181 [p-tau(181)] were measured using Fujirebio Lumipulse or Innotest assay as described (Lehmann et al. 2020). The cutoff values were initially obtained from groups of patients clinically diagnosed with AD (whose clinical diagnoses were made blind to biomarker results) and, for the Barcelona cohort, from amyloid-PET positive and amyloid-PET negative participants (Alcolea et al. 2019, PMID 31464088) or, for Montpellier, from control population of the memory clinic with various etiology (Lehmann et al. 2013, 2018). Based on these data, a nonpathological CSF profile corresponding the (A−/T−/N−) situation is defined as having a value of the Aβ_1–42_/Aβ_1–40_ ratio (*A*) above the cutoff and values of tau (*N*) and p-tau(181) (*T*) below the pathological cutoffs. We also identified amyloid (A+/A−) and tau-neurodegeneration (N+T+/N−T−) CSF profiles.

Three different approaches were used to measure plasma levels of Aβ_1–42_ and Aβ_1–40_: “Neurology 3-Plex A” (Q3, both cohorts) and “Neurology 4-plex E Advantage kit” (Q4, Montpellier cohort) in the Simoa platform (Quanterix) and an IP-MS approach from Shimadzu (Nakamura et al. 2018) (both cohorts) implemented in Montpellier’s laboratory and slightly modified from the original protocol (Alcolea et al. 2021). Levels of p-tau(181) were measured in the Simoa platform (Quanterix).

### Statistical analysis

Statistical analyses were completed with Medcalc (v19.8). The accuracy of the blood-based assays to discriminate nonpathological amyloid and tau CSF profile was evaluated using receiver operating characteristic (ROC) curve analysis and calculation, using the area under the curve (AUC) as a measure of diagnostic accuracy. Comparison of ROC curves to test the statistical significance between assay values derived from the method of DeLong et al. (1988) for the calculation of the standard error of the AUCs. Multiple regression was used to examine the relationship between CSF and blood assays allowing to combine them and evaluate if they were independent or not. Logistic regression used to combine independent factors was employed using different ways of introducing the factors into the algorithm (Enter/Forward/Backward/Stepwise) if their *p* values were < 0.05 and removed if *p* > 0.1.

### Results

We first tested the performance (AUC) of blood biomarkers to distinguish a nonpathological (A−/T−/N−) from a pathological CSF profile represented by the other A/T/N situations (Table 1). Plasma Aβ_1–40_ and Aβ_1–42_, individually, show variable but low accuracy for nonpathological profiles detection (Table 2 and Supp Table 1). In contrast, the Aβ_1–42_/Aβ_1–40_ ratio showed much higher performance regardless of the analytical method used (Q3, Q4 or IP-MS).

**Table 2:** Diagnostic accuracy of plasma biomarkers to discriminate non pathological CSF (A−/T−/N−) profiles in the cohort of Montpellier and Barcelona

Non-pathological CSF (A−/T−/N−)	The Montpellier cohort				The Barcelona cohort			
Blood biomarkers	AUC	SE	95% CI	*p*	AUC	SE	95% CI	*p*
Aβ_1–40(Q3)_	0.661	0.069	0.530–0.777	**0.020**	0.638	0.080	0.490–0.769	0.085
Aβ_1–40(Q4)_	0.672	0.068	0.542–0.785	**0.011**	–	–	–	–
Aβ_1–40(IP-MS)_	0.526	0.074	0.396–0.653	0.728	0.549	0.051	0.463–0.633	0.329
Aβ_1–42(Q3)_	0.548	0.077	0.417–0.675	0.534	0.715	0.076	0.571–0.834	**0.044**
Aβ_1–42(Q4)_	0.542	0.076	0.410–0.669	0.601	–	–	–	–
Aβ_1–42(IP-MS)_	0.638	0.073	0.507–0.755	0.060	0.656	0.048	0.571–0.733	**0.001**
Tau	0.612	0.078	0.480–0.733	0.149	0.618	0.081	0.470–0.752	0.495
p-tau(181)	0.865	0.049	0.756––0.938	**< 0.0001**	0.773	0.039	0.697–0.837	**< 0.0001**
Aβ_1–42_/Aβ_40(Q3)_	0.709	0.068	0.580–0.818	**0.002**	0.848	0.062	0.719–0.934	**< 0.0001**
Aβ_1–42_/Aβ_40(Q4)_	0.753	0.065	0.627–0.854	**< 0.0001**	–	–	–	–
Aβ_1–42_/Aβ_40(IP-MS)_	0.715	0.066	0.587–0.822	**0.001**	0.661	0.048	0.577–0.739	**0.0008**
Logistic regression Aβ_1–40_, Aβ_1–42_, p-tau(181)	0.904	0.040	0.804–0.964	**< 0.0001**	0.882	0.050	0.759–0.956	**< 0.0001**

Biomarkers were quantified using either Quanterix technology (Q3 and Q4) or Shimadzu approach (IP-MS). Significant differences are indicated by bolded *p* (threshold 0.05)

**Fig. 1:**
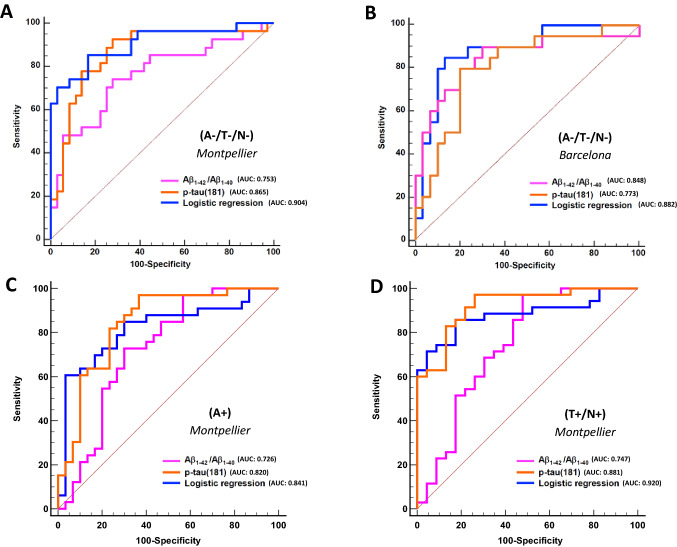
Receiver operating characteristic (ROC) curves for plasma biomarkers to discriminate non pathological (A−/T−/N−) (**A** and **B**), amyloid (A+) (**C**) or neurodegenerative (T+/N+) (**D**) CSF profiles. Lines indicate areas under the curve (AUC) for individual biomarker (orange) or ratios (pink) to discriminate CSF profiles. Blue line corresponds to the ROC curve yielded by a logistic regression that included all three plasma markers and ratios

In both cohorts, plasma levels of total tau were comparable between nonpathological and pathological CSF profiles, while elevated plasma p-tau(181) was associated with a pathological CSF profile (Supp Table 1). Plasma p-tau(181) showed an AUC of 0.865 in Montpellier and 0.773 in Barcelona to discriminate a nonpathological (A−/T−/N−) CSF profile (Table 1). Pairwise comparison of AUCs confirmed the high performance of p-tau(181) when compared to Aβ_1–42_ and total tau (*p* < 0.05) in Montpellier cohort. In Barcelona cohort, AUCs obtained for p-tau(181), Aβ_1–42(Q3)_ and Aβ_1–42(IP-MS)_ were similar (Table 2) and AUC of p-tau(181) was significantly higher than AUC of total tau (*p* = 0.022).To assess the value of combining biomarkers, we first tested the correlation between the different factors and observed that Aβ_1–42_, Aβ_1–40_, Aβ_1–42_/Aβ_1–40_ on the one hand, and tau, p-tau(181) on the other hand, were correlated together (Pearson correlation; *p* < 0.001). We therefore selected as independent variable Aβ_1–42_/Aβ_1–40_ and p-tau(181) (with amyloid peptides measured with Q4 and Q3 Quanterix in Montpellier and Barcelona, respectively) that had the best AUCs. Combining biomarkers requires a stepwise approach (Mamtani et al. 2006), however in our case we only have two factors and we tested different logistic regression approaches (see “Methods”) which all resulted in the same algorithm confirming the independence and statistical relevance of the two selected biomarkers. As illustrated (Table 2 and Fig. 1A), AUCs obtained for Aβ_1–42_/Aβ_1–40_ (both measured with Quanterix Q4), p-tau(181) and logistic regression combining these three parameters were very close and pairwise comparison of the different AUCs was significant only in Montpellier cohort between Aβ_1–42_/Aβ_1–40_ and logistic regression (Supp Table 3).

The highest Youden index in this context was obtained when performing the logistic regression, reaching a sensitivity of 85.2% and a specificity of 83.6% for the detection of nonpathological amyloid and tau CSF profiles. In the Barcelona cohort (Table 1 and Fig. 1B), pairwise comparison was not significant between p-tau(181) and Aβ_1–42_/Aβ_1–40_ (measured with Quanterix Q3). However, the AUC of logistic regression was higher than that of p-tau(181) alone (*p* = 0.002). In this cohort, the highest Youden index was obtained when performing the logistic regression, reaching a sensitivity of 85.0% and a specificity of 86.7% for the detection of nonpathological amyloid and tau CSF profiles.

We also studied the performance of biomarkers to identify two specific pathological situations with amyloid (A+, Sup Table 1, Fig. 1C) or tau-neurodegeneration (T+/N+, Sup Table 2, Fig. 1D) profiles. p-tau(181) and logistic regression were again the most discriminant. The performance of the Aβ_1–42_/Aβ_1–40_ ratio was comparatively lower, especially for the T+/N+ profile.

## Discussion

An important stage in the management of patients consulting for cognitive complaints is the decision to perform or not a LP, which likely provides early indicators of neurodegenerative diseases. Indeed, CSF analysis provides indirect signs to a broader range of diagnoses than AD since amyloid and tau biomarkers might also be altered in different pathological situations such as non-Alzheimer's neurodegenerative diseases like DLB, FTLD and Creutzfeldt-Jakob disease (Gabelle et al. 2011; Bousiges et al. 2018; Bibl et al. 2008; Lehmann et al. 2019) as well as brain damage (Alosco et al. 2018), normal pressure hydrocephalus (Manniche et al. 2020) and cerebral amyloid angiopathy (Renard et al. 2012) ^19^. This is illustrated in our cohorts by the fact that non-AD patients represent 40–50% of the CSF pathological profiles.

In this study, we evaluated the performance of blood biomarkers for the detection of “nonpathological” (A−/T−/N−), amyloid (A+) or neurodegenerative (T+/N+) CSF profiles. The main interest of the detection of a nonpathological (A−/T−/N−) profile lies in the fact that this information can be taken into account in the decision to perform a LP or not. Using different analytical approaches and in two independent cohorts, we show that plasma p-tau(181) and Aβ_1–42_/Aβ_1–40_ ratio achieve the best performance to detect nonpathological CSF profiles. Interestingly, the amyloid ratio performed better in the Barcelona cohort, which may be explained by the fact that this cohort has a significantly higher percentage of positive amyloid profiles (Table 1). These plasma biomarkers are also those identified as the best predictors of AD (Palmqvist et al. 2021), but here, the context of use is different, and their performance are event higher than for discriminating AD from non-AD. Other differences are noted such as the fact that Aβ_1–42_ detection by IP-MS outperformed other Aβ_1–42_ detections, but this was not the case when considering the Aβ_1–42_/Aβ_1–40_ ratio, thus differently than when AD is the performance criterion (Janelidze et al. 2021). As mentioned above, this could be partly explained by the fact that diseases other than AD showed pathological amyloid or tau profiles. Thus, combining plasma amyloid and p-tau(181) slightly increased the performance, therefore suggesting their complementarity. This was confirmed when the blood biomarker performance criteria were based on the detection of amyloid (A+) or neurodegenerative (T+/N+) CSF profiles (Fig. 1). Strikingly, blood p-tau(181) outperforms the amyloid ratio for the detection of an amyloid profile in CSF. This confirms the value of detecting phosphorylated tau proteins in blood. In this work we quantified blood p-tau(181) but other phosphorylated isoforms, such as p-tau(217) or p-tau(231) which have shown better diagnostic performance (Brickman et al. 2021; Barthelemy et al. 2020; Bayoumy et al. 2021), can be expected to be even more effective.

With performances close to 85% sensitivity and specificity for the detection of nonpathological (A−/T−/N−) CSF profiles, one can really consider the results of blood biomarkers, which would then condition the subsequent need for a LP. Importantly, the biomarker cutoff decision points will likely be different than for AD detection. Such an approach could help clinicians in the decision to add other diagnostic tests (such as imaging), depending on the clinical evaluation of the patient. Depending on the prevalence of AD as well as that of other diseases modifying CSF amyloid and tau concentrations in a cohort, the reduction in the need for LP could be well over 50%, significantly reducing the cost of management of these patients and limiting invasive and unnecessary medical procedures. In conclusion, blood amyloid and tau biomarkers perform well in detecting nonpathological amyloid and tau CSF patterns. The importance and value of this “prediction” are linked to the exclusion of pathologies that vary these biomarkers (AD but not only) and to the decision to perform a LP or not. Blood biomarkers can therefore represent the first step in the patient's management strategy, to determine whether or not other diagnostic examinations by more invasive or more expensive means are necessary.

## Supplementary Information

Below is the link to the electronic supplementary material.

Supplementary file1 (DOCX 28 kb)
