# Supplementary material for: Blood amyloid and tau biomarkers as predictors of cerebrospinal fluid profiles
Source: J Neural Transm (Vienna). 2022 Feb 15;129(2):231–7. doi: 10.1007/s00702-022-02474-9 (PMC8866346; doi:10.1007/s00702-022-02474-9)
Supplement: Supplementary file 1 — Supplementary file1 (DOCX 28 kb) [file 702_2022_2474_MOESM1_ESM.docx]

# ****SUP TABLE 1****

|  | **The Montpellier cohort** | | | | | | **The Barcelona cohort** | | | | | |
| --- | --- | --- | --- | --- | --- | --- | --- | --- | --- | --- | --- | --- |
|  | **Non-pathological (A-/T-/N-) CSF** |  | **Pathological CSF** |  |  | **Non-pathological (A-/T-/N-) CSF** | |  | **Pathological CSF** |  |  |  |
| **Blood Biomarkers** | Mean | SD | Mean | SD | P | Mean | | SD | Mean | SD | P |  |
| **Aβ_1-40(Q3)_** | 259.636 | 82.479 | 215.476 | 49.235 | **0.018** | 225.890 | | 69.730 | 200.782 | 33.602 | 0.141 |  |
| **Aβ_1-40(Q4)_** | 136.096 | 40.076 | 115.027 | 25.763 | **0.020** | / | | / | / | / | / |  |
| **Aβ_1-40(IP-MS)_** | 30.066 | 9.925 | 29.272 | 8.092 | 0.735 | 1.067 | | 0.353 | 1.101 | 0.356 | 0.573 |  |
|  | | | | | | |  | | | | | |
| **Aβ_1-42(Q3)_** | 12.601 | 3.473 | 12.584 | 3.040 | 0.984 | 7.577 | | 2.283 | 8.722 | 1.649 | **0.045** |  |
| **Aβ_1-42(Q4)_** | 6.264 | 1.902 | 6.4485 | 1.644 | 0.688 | / | | / | / | / | / |  |
| **Aβ_1-42(IP-MS)_** | 0.508 | 0.122 | 0.572 | 0.139 | 0.056 | 0.047 | | 0.014 | 0. 041 | 0.017 | **0.023** |  |
|  | | | | | | |  | | | | | |
| **Tau** | 2.187 | 0.8207 | 1.851 | 1.114 | 0.176 | 2.121 | | 0.990 | 2.557 | 1.566 | 0.229 |  |
| **p-tau (181)** | 1.783 | 0.633 | 0.969 | 0.536 | **<0.0001** | 15.958 | | 15.659 | 23.410 | 10.465 | **0.002** |  |
|  | | | | | | |  | | | | | |
| **Aβ_1-42_/_40 (Q3)_** | 0.051 | 0.011 | 0.059 | 0.013 | **0.005** | 0.034 | | 0.005 | 0.044 | 0.008 | **<0.0001** |  |
| **Aβ_1-42_/_40 (Q4)_** | 0.0467 | 0.009 | 0.057 | 0.010 | **0.0002** | / | | / | / | / | / |  |
| **Aβ_1-42_/_40 (IP-MS)_** | 0.0184 | 0.008 | 0.0199 | 0.003 | 0.341 | 0.045 | | 0.010 | 0.039 | 0.012 | **0.008** |  |

# Supplemental table 1: Accuracy of plasma biomarkers to discriminate non pathological CSF profiles in the cohort of Montpellier and Barcelona. Biomarkers were quantified using either Quanterix technology (Q3 and Q4) or IP-MS approach (IP-MS). Results are indicated as mean and standard deviation. Significative differences are indicated by bolded p (threshold 0.05).

**SUP TABLE 2**

| **Amyloid positive (A+) CSF** | **The Montpellier cohort** | | | |  | **The Barcelona cohort** | | | |
| --- | --- | --- | --- | --- | --- | --- | --- | --- | --- |
| **Blood Biomarkers** | **AUC** | **SE** | **95% CI** | **p** |  | **AUC** | **SE** | **95% CI** | **p** |
| **Aβ_1-40(Q3)_** | 0.564 | 0.074 | 0.432 to 0.690 | 0.388 |  | 0.629 | 0.080 | 0.481 to 0.761 | 0.108 |
| **Aβ_1-40(Q4)_** | 0.606 | 0.072 | 0.475 to 0.727 | 0.141 |  | / | / | / | / |
| **Aβ_1-40(IP-MS)_** | 0.504 | 0.074 | 0.375 to 0.633 | 0.957 |  | 0.552 | 0.051 | 0.466 to 0.636 | 0.308 |
|  |  |  |  |  |  |  |  |  |  |
| **Aβ_1-42(Q3)_** | 0.574 | 0.075 | 0.442 to 0.699 | 0.322 |  | 0.689 | 0.080 | 0.542 to 0.812 | **0.018** |
| **Aβ_1-42(Q4)_** | 0.555 | 0.074 | 0.424 to 0.680 | 0.457 |  | / | / | / | / |
| **Aβ_1-42(IP-MS)_** | 0.676 | 0.068 | 0.547 to 0.789 | **0.010** |  | 0.604 | 0.049 | 0.518 to 0.685 | **0.037** |
|  |  |  |  |  |  |  |  |  |  |
| **Tau** | 0.579 | 0.074 | 0.447 to 0.703 | 0.289 |  | 0.524 | 0.082 | 0.381 to 0.664 | 0.774 |
| **p-tau(181)** | 0.820 | 0.056 | 0.703 to 0.906 | **<0.0001** |  | 0.731 | 0.042 | 0.652 to 0.800 | **<0.0001** |
|  |  |  |  |  |  |  |  |  |  |
| **Aβ_1-42_/ Aβ_40(Q3)_** | 0.662 | 0.071 | 0.531 to 0.778 | **0.021** |  | 0.703 | 0.087 | 0.521 to 0.822 | **0.019** |
| **Aβ_1-42_/ Aβ_40(Q4)_** | 0.726 | 0.067 | 0.599 to 0.831 | **0.001** |  | / | / | / | / |
| **Aβ_1-42_/ Aβ_40(IP-MS)_** | 0.728 | 0.065 | 0.601 to 0.833 | **0.001** |  | 0.613 | 0.049 | 0.527 to 0.694 | **0.022** |
|  |  |  |  |  |  |  |  |  |  |
| **Logistic regression Aβ_1-40_, Aβ_1-42_, p-tau(181)** | 0.841 | 0.052 | 0.728 to 0.921 | **<0.0001** |  | 0.816 | 0.060 | 0.681 to 0.911 | **<0.0001** |

**Sup Table 2: Diagnostic accuracy of plasma biomarkers to discriminate amyloid positive (A+) vs negative (A-) CSF profiles in the cohort of Montpellier and Barcelona.** Biomarkers were quantified using either Quanterix technology (Q3 and Q4) or Shimadzu approach (IP-MS). Significant differences are indicated by bolded p (threshold 0.05).

**SUP TABLE 3**

| **Tau-Neurodegeneration positive (N+/T+) CSF** | **The Montpellier cohort** | | | |  | **The Barcelona cohort** | | | |
| --- | --- | --- | --- | --- | --- | --- | --- | --- | --- |
| **Blood Biomarkers** | **AUC** | **SE** | **95% CI** | **p** |  | **AUC** | **SE** | **95% CI** | **p** |
| **Aβ_1-40(Q3)_** | 0.603 | 0.078 | 0.464 to 0.730 | 0.186 |  | 0.573 | 0.091 | 0.415 to 0.721 | 0.420 |
| **Aβ_1-40(Q4)_** | 0.651 | 0.072 | 0.514 to 0.771 | **0.037** |  | / | / | / | / |
| **Aβ_1-40(IP-MS)_** | 0.514 | 0.078 | 0.379 to 0.648 | 0.855 |  | 0.565 | 0.053 | 0.474 to 0.652 | 0.227 |
|  |  |  |  |  |  |  |  |  |  |
| **Aβ_1-42(Q3)_** | 0.574 | 0.081 | 0.436 to 0.704 | 0.362 |  | 0.808 | 0.069 | 0.662 to 0.911 | **<0.0001** |
| **Aβ_1-42(Q4)_** | 0.537 | 0.079 | 0.401 to 0.669 | 0.636 |  | / | / | / | / |
| **Aβ_1-42(IP-MS)_** | 0.684 | 0.074 | 0.549 to 0.800 | **0.013** |  | 0.632 | 0.052 | 0.542 to 0.715 | **0.012** |
|  |  |  |  |  |  |  |  |  |  |
| **Tau** | 0.597 | 0.081 | 0.459 to 0.725 | 0.232 |  | 0.636 | 0.087 | 0.477 to 0.775 | 0.120 |
| **p-tau(181)** | 0.881 | 0.045 | 0.770 to 0.951 | **<0.0001** |  | 0.785 | 0.041 | 0.707 to 0.851 | **<0.0001** |
|  |  |  |  |  |  |  |  |  |  |
| **Aβ_1-42_/ Aβ_40(Q3)_** | 0.679 | 0.076 | 0.542 to 0.797 | **0.020** |  | 0.843 | 0.067 | 0.709 to 0.932 | **<0.0001** |
| **Aβ_1-42_/ Aβ_40(Q4)_** | 0.747 | 0.072 | 0.615 to 0.852 | **0.002** |  | / | / | / | / |
| **Aβ_1-42_/ Aβ_40(IP-MS)_** | 0.773 | 0.062 | 0.644 to 0.872 | **<0.0001** |  | 0.639 | 0.052 | 0.550 to 0.723 | **0.007** |
|  |  |  |  |  |  |  |  |  |  |
| **Logistic regression Aβ_1-40_, Aβ_1-42_, p-tau(181)** | 0.920 | 0.035 | 0.819 to 0.975 | **<0.0001** |  | 0.911 | 0.050 | 0.786 to 0.976 | **<0.0001** |

**Sup Table 3: Diagnostic accuracy of plasma biomarkers to discriminate Tau-Neurodegeneration (N+/T+) vs negative (N-/T-) CSF profiles in the cohort of Montpellier and Barcelona.** Biomarkers were quantified using either Quanterix technology (Q3 and Q4) or Shimadzu approach (IP-MS). Significant differences are indicated by bolded p (threshold 0.05).
